# Vegetation–climate feedbacks across scales

**DOI:** 10.1111/nyas.15286

**Published:** 2025-01-24

**Authors:** Diego G. Miralles, Jordi Vilà‐Guerau de Arellano, Tim R. McVicar, Miguel D. Mahecha

**Affiliations:** ^1^ Hydro‐Climate Extremes Lab (H‐CEL) Ghent University Ghent Belgium; ^2^ Meteorology and Air Quality Group Wageningen University & Research Wageningen Netherlands; ^3^ CSIRO Environment Canberra Australian Capital Territory Australia; ^4^ Australian Research Council Centre of Excellence for Climate Extremes Canberra Australian Capital Territory Australia; ^5^ Institute for Earth System Science and Remote Sensing Leipzig University Leipzig Germany; ^6^ Helmholtz Centre for Environmental Research UFZ Leipzig Germany; ^7^ German Centre for Integrative Biodiversity Research (iDiv) Leipzig‐Halle‐Jena Germany

**Keywords:** climate extremes, droughts and heatwaves, land–atmosphere interactions, vegetation–climate feedbacks

## Abstract

Vegetation is often viewed as a consequence of long‐term climate conditions. However, vegetation itself plays a fundamental role in shaping Earth's climate by regulating the energy, water, and biogeochemical cycles across terrestrial landscapes. It exerts influence by consuming water resources through transpiration and interception, lowering atmospheric CO_2_ concentration, altering surface roughness, and controlling net radiation and its partitioning into sensible and latent heat fluxes. This influence propagates through the atmosphere, from microclimate scales to the entire atmospheric boundary layer, subsequently impacting large‐scale circulation and the global transport of heat and moisture. Understanding the feedbacks between vegetation and atmosphere across multiple scales is crucial for predicting the influence of land use and land cover changes, and for accurately representing these processes in climate models. This review discusses the biophysical and biogeochemical mechanisms through which vegetation modulates climate across spatial and temporal scales. Particularly, we evaluate the influence of vegetation on circulation patterns, precipitation, and temperature, considering both long‐term trends and extreme events, such as droughts and heatwaves. Our goal is to highlight the state of science and review recent studies that may help advance our collective understanding of vegetation feedbacks and the role they play in climate.

## Graphical abstract

Vegetation does not merely respond to climate: it actively shapes it. Plants cool and moisten the air, regulate carbon dioxide levels, alter sunlight reflection, and influence winds. These impacts cascade from microclimates to planetary scales, altering weather events and climate trends. Our review synthesizes recent advances in understanding vegetation–climate interactions, while identifying critical knowledge gaps that need to be addressed to improve climate prediction and develop effective adaptation strategies.

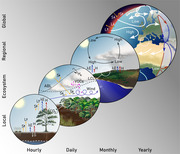

## INTRODUCTION

For centuries, the interplay between climate and vegetation has captivated scientists. It is a relationship of give and take: while vegetation relies on the environment for survival, it also plays a crucial role in shaping Earth's climate. Through a variety of biophysical and biogeochemical processes, vegetation controls the flow of energy, water, carbon, and other chemicals in the critical zone of our planet. It modulates wind patterns, moistens the air via transpiration and interception loss, regulates atmospheric carbon dioxide (CO_2_) concentration, and controls surface net radiation and its partitioning into latent and sensible heat fluxes. The study of this interplay can be traced back to Alexander von Humboldt, who in 1850 theorized that vegetation loss could alter local climate patterns, reduce rainfall, and disrupt ecosystem dynamics.^1^ His research journeys laid the groundwork for substantial scientific advancements, including Vladimir Vernadsky's 1926 vision of the biosphere playing an active role in shaping the Earth's biogeochemical cycles,^2^ and Wladimir Köppen's 1936 idea that climatic boundaries are the main drivers of biogeographical patterns.^3^ However, it was not until the 1980s, with the development of coupled climate models which encoded vegetation and soil processes, that the meteorology and climatology communities fully recognized the central role of plants in the climate system.^4^, ^5^ These models required a mathematical representation of land fluxes regulated by vegetation, which did not conform to the mathematics of fluid dynamics that were used to represent the atmosphere. Thereby, even the most traditional atmospheric scientists started to acknowledge the undeniable influence of terrestrial ecosystems on atmospheric processes, and the need to represent plant behavior accurately to predict upcoming weather and future climate.^6^

Today, it is understood that the impact of vegetation on the climate system is far‐reaching, affecting every scale, from local microclimates to global atmospheric circulation, influencing the severity of meteorological extremes, and shaping long‐term climate trends.^7^, ^8^, ^9^ As such, understanding vegetation–atmosphere feedbacks across spatiotemporal scales is essential to anticipate how the biosphere's response and adaptation to climate change and land use will, in turn, influence future climate.^10^ Despite the recognition of its crucial importance since the 1980s, recent reports by the Intergovernmental Panel on Climate Change (IPCC) have only briefly addressed vegetation feedbacks, with the biophysical ones (e.g., those related to leaf area, albedo, roughness, and transpiration) remaining particularly understudied.^11^ This gap partly reflects a historical focus on atmospheric feedbacks—such as the cloud, lapse rate, and water vapor feedbacks—which are, nonetheless, also influenced by vegetation state and activity. Furthermore, challenges to accurately model biophysical processes in climate models continue to limit our understanding.^9^, ^10^

In this review, we discuss the biophysical and biogeochemical mechanisms through which vegetation influences climate at various spatiotemporal scales, examining its impact on energy, water, carbon, and momentum fluxes, and their myriad of linkages to atmospheric boundary layer (ABL) thermodynamics, mesoscale and synoptic circulation, and ultimately global precipitation, temperature, and humidity patterns. Furthermore, we review the climatic consequences of vegetation changes, with specific emphasis on extreme events like droughts and heatwaves. In doing so, we synthesize the current understanding of vegetation–climate feedbacks and highlight recent studies that have advanced knowledge on the role of vegetation in our climate system.

## PLANT CONTROL OVER ENERGY, WATER, AND CARBON FLUXES

To review the different pathways by which plants exert control over the state of the atmosphere, the combination of the surface radiation budget and the energy balance equation provides an excellent foundation:

(1) $$R_{n}=S↓-\;S↑+\;L↓-\;L↑=\lambda E+H+G+\left[\text{…}\right],$$

where R_n_ represents surface net radiation, S↓ and S↑ are the incoming and outgoing shortwave radiation, respectively, and L↓ and L↑ are the longwave counterparts, which depend on atmospheric and land surface temperature, respectively, following the Stefan–Boltzmann law. λE is the latent heat flux associated with evaporation, H is the sensible heat flux, and G is the ground heat flux. λE and H are turbulent fluxes that depend on gradients between surface and atmospheric properties, while G is controlled by the vertical gradient of temperature (and moisture) in the soil.^12^ All these fluxes are typically expressed in units of W m^−2^. More complex versions of this equation include terms such as advective energy, the conversion of momentum into thermal energy, and/or the energy associated with temporal changes in net ecosystem carbon exchange, thereby the “[…]” at the end of the equation. The magnitude of these secondary terms depends on the ecosystem but is typically negligible over large temporal and spatial scales, with R_n_ being partitioned almost entirely between λE and H.^13^

**FIGURE 1:**
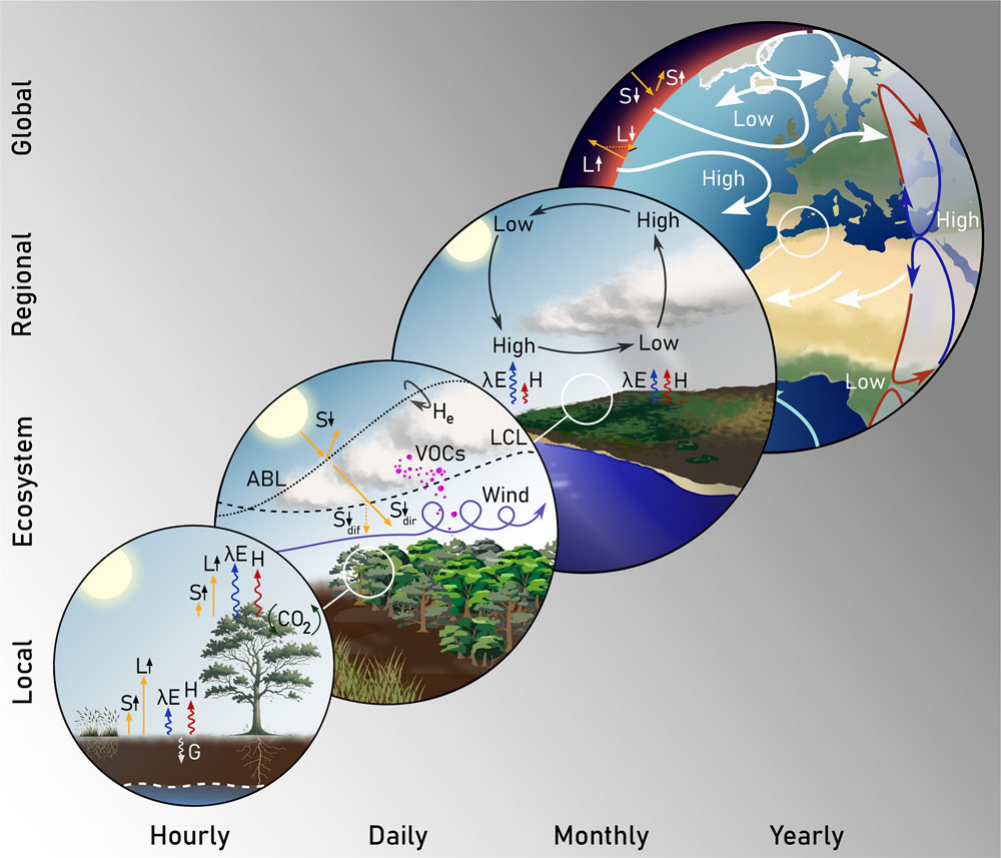
Influence of vegetation on the atmosphere across meteorological scales. Vegetation controls the surface energy, water, and carbon fluxes at local scales, influenced by soil moisture and the dynamics of the groundwater table. This influence propagates to the atmosphere via turbulent fluxes, causing convective and mechanical instability, altering the state and diurnal growth of the atmospheric boundary layer (ABL), regulating moisture and heat entrainment (H_e_), and thus the lifting condensation level (LCL) and convective cloud formation. At larger scales, vegetation influences mesoscale circulation and the location of semi‐permanent low‐ and high‐pressure systems, affecting the redistribution of heat, water, and carbon and thus influencing temperature, humidity, and precipitation patterns at planetary scales.

A key influence of vegetation on climate comes from the degree to which the plant concentration of pigments and its structural properties, such as the leaf area index (LAI) and leaf angles, modify the albedo, and thus how much of the incoming (direct and diffuse) shortwave radiation (S↓) is absorbed (and contributes to R_n_) versus how much is reflected (S↑). The albedo of ecosystems is dynamic, varying in both time and space. High‐albedo ecosystems include snow‐covered landscapes and deserts, whereas forests typically have a low albedo, absorbing more radiation (Figure 1). Besides those related to solar incidence angles, temporal changes in surface albedo reflect phenological and disturbance dynamics, ecosystem transformations due to land use change, and ecological succession. These processes thus play a role in regional energy balances and the climate system as a whole. Moreover, the temperature of an ecosystem, and, therefore, its L↑, is directly affected by the thermal properties of vegetation, its albedo, and its evaporation rate (λE). On average, healthy and unstressed vegetated ecosystems warm more slowly during the day, resulting in lower L↑, which is inversely related to vegetation density (Figure 1).

Vegetation's control upon R_n_ extends beyond outgoing radiative fluxes (S↑, L↑); plants also influence incoming radiation (S↓, L↓) through complex interactions with the ABL that control convective cloud formation, for instance, via emission of biogenic volatile organic compounds (BVOCs) that act as aerosol precursors and can form cloud droplets, and through the plants’ regulation of atmospheric CO_2_, water vapor (H_2_O), and methane (CH_4_) concentration (Figure 1). By absorbing CO_2_ during photosynthesis, which is then released over various time scales through respiration, ecosystems modulate the greenhouse effect of our planet and thus L↓. Moreover, vegetation indirectly regulates CH_4_ production and oxidation by controlling soil moisture and temperature, and emerging research suggests that trees directly produce and uptake CH_4_.^14^ This influence on the global carbon cycle through CO_2_ and CH_4_ fluxes is crucial to Earth's greenhouse gas budget, affecting the global energy balance, temperature, and long‐term climate patterns, as shown in paleoclimatic records.^15^ In fact, the enhanced photosynthesis and subsequent greening as a consequence of CO_2_ fertilization, global warming, and nutrient deposition in recent decades has led to the sinking of approximately one‐third of anthropogenic CO_2_ emissions.^16^ The consequent dampening of the secondary greenhouse effect in our planet implies a negative biogeochemical feedback on temperature that has been quantified as approximately –0.8 W m^−2^°C^−1^.^15^ In addition to CO_2_, CH_4_, and H_2_O, vegetation also influences the concentration of other potent greenhouse gasses, such as nitrous oxide (N_2_O) and ozone (O_3_).^17^

Plants do not only affect all four radiative fluxes in Equation (1), but also the partitioning of R_n_ among λE, H, and G. By reducing land surface temperature and thermal conductance, vegetated ecosystems typically experience lower G than their surroundings.^6^ This lower G is also a consequence of how the physical structure and density of vegetation enhance the tendency of the near‐surface atmosphere to conduct and convect mass and heat, favoring the dissipation of R_n_ into the atmosphere via turbulent fluxes (λE and H). In dense forests with tall trees, surface roughness creates a drag on the airflow, reducing average wind speed but enhancing turbulence within and just above the canopy. The net effect is increased aerodynamic conductance, enhancing the exchange of momentum, heat, and mass from land to atmosphere (Figure 1). Recent studies show the key relevance of accurately representing aerodynamic conductance within the canopy layer in climate models,^18^ and attribute the influence of greening on climate mostly to increases in aerodynamic conductance.^19^

The partitioning of available energy (AE = R_n_– G) between λE and H is also dynamically controlled by vegetation and varies spatiotemporally across ecosystems. In forested areas, when sufficient soil moisture is available and energy‐limited conditions prevail, a higher proportion of AE is converted into λE, leading to a cooling effect on the local climate (Figure 1). This phenomenon is attributed to the extensive leaf area and deep roots of forests, which lead to high water uptake and loss through transpiration, but also to an intense flux of interception loss during and after periods of rain. The water intercepted by plants and subsequently evaporated without reaching the soil can have crucial importance for humidity, fog, and cloud formation, and has complex implications for the energy balance of forested ecosystems.^20^ Conversely, regions characterized by sparse vegetation and low transpiration, such as drylands, predominantly channel R_n_ into H, which contributes to warmer atmospheric conditions. This dynamic partitioning of AE between the two turbulent fluxes affects local weather patterns, atmospheric stability, and precipitation processes, and has been at the core of the study of the ecosystem's influence on the atmosphere for at least a century.^21^

Ultimately, plants form a nexus among the energy, carbon, and water cycles. Through their transpiration and rainfall interception, vegetation links energy and water cycles, not only cooling but also moistening the surface layer of the atmosphere and affecting humidity and cloud formation in the ABL (see next section). Likewise, vegetation connects energy and carbon cycles through the consumption of photosynthetically active radiation (PAR) during photosynthesis, and through the uptake and release of CO_2_ that controls L↓. Finally, photosynthesis and transpiration—and, therefore, carbon, water, and energy cycles—are intrinsically linked through the stomata openings on the leaf, which regulate the exchange of CO_2_, water vapor, and oxygen with the atmosphere.^22^ Stomatal conductance is influenced by environmental factors such as PAR, temperature, and vapor pressure deficit (VPD), which also depend on vegetation–atmosphere feedbacks, and its optimization reflects the delicate balance plants maintain to maximize carbon gain while preventing dehydration.^23^ This interconnection ensures cascading effects among cycles, emphasizing the need for holistic approaches to understand how plants (and the ecosystems they sustain) shape atmospheric conditions across scales.^7^

## INFLUENCE OF VEGETATION ON LOCAL ATMOSPHERIC CONDITIONS

The influence of vegetation on the local atmosphere extends from microscale interactions at the stomata to ABL dynamics. The biophysical and biogeochemical processes described in the previous section affect the diurnal variability and profiles of humidity and temperature, ABL growth, and atmospheric thermodynamic stability. A key process governing the exchange of heat, water, and carbon in the land–atmosphere interface is turbulence. Turbulence can either be mechanically generated by wind instabilities or triggered by air density gradients, with both being directly controlled by surface roughness and energy partitioning, and thus by vegetation's presence, structure, state, and activity. As such, vegetation affects the formation of convective clouds and rainfall, and, therefore, also the partitioning of S↓ between diffuse and direct radiation, which is essential for photosynthesis itself (Figure 1).

The study of the impact of the land surface on the ABL can be traced back to the mid‐20th century,^24^ yet the field gained significant stimulus in the latter half, leveraging advances in computer and data sciences, instrumentation (e.g., eddy covariance), and theoretical understanding. In the 1970s and 1980s, the advent of more sophisticated land–surface schemes in numerical weather prediction (and observational techniques, such as remote sensing) allowed for a more detailed examination of vegetation influences on ABL (thermo)dynamics. Large eddy simulation (LES) models also emerged as powerful computational tools for explicitly studying turbulent flows.^25^ However, it was not until the late 20th century that LES was employed for the first time to study ABL dynamics and their dependence on terrestrial ecosystems.^26^

Today, we understand that the stability and growth of the ABL depends on boundary conditions such as subsidence, advection, and the properties of the residual and free tropospheric layers. However, the diurnal development of the ABL is first and foremost triggered by surface turbulent fluxes (regulated by vegetation) and their influence on the entrainment of air from the residual layer and free troposphere as the ABL grows. In dry conditions and bare (or sparsely vegetated) lands, limited transpiration results in enhanced H and a warming of the near‐surface layer. This often creates a strong thermal instability that promotes the rapid growth of the ABL. Nonetheless, the absence of moisture often yields a drier and warmer ABL, which can be associated with reduced cloud formation unless moisture is entrained or advected from elsewhere.^27^ Conversely, the presence of dense and active vegetation allows for enhanced transpiration and interception loss, contributing to cooling the surface and adding moisture to the air (Figure 1). A reduced H may favor the development of more stable, moist‐convective boundary layers, which tend to grow more slowly than those over bare soils. However, the lower albedo, higher moisture content, strong mechanical turbulence, and BVOC emission may still lead to preferential cloud formation over forested regions compared to their surroundings.^28^

The diurnal growth of the ABL is ultimately enabled by the entrainment of air and the advection of air masses.^29^ Entrained air, with residual characteristics from previous days or free tropospheric conditions, influences the state of the ABL and the near‐surface atmosphere (Figure 1). In the morning, the air entrained as the ABL grows is relatively warm and dry^30^ and has a lower CO_2_ concentration.^31^ This air enhances VPD at the canopy level, and leads to increased evaporation, thus shifting the turbulent heat flux partitioning toward λE. This effect can be partly offset by the closure of stomata to down‐regulate plant transpiration in response to higher VPD under limited soil water availability. During the day, CO_2_ concentration decreases due to the combined effects of entrainment and plant CO_2_ uptake for photosynthesis, albeit partly offset by ecosystem respiration.^31^, ^32^ This dynamic interplay between vegetation and ABL during the day is crucial for the formation and intensification of boundary‐layer clouds, such as shallow cumuli.^7^, ^33^ Moreover, modifications of vegetation activity by the combined effects of cloud shading, and changes in the temperature, CO_2_ concentration, and VPD lead to shifts in the R_n_ partitioning between H and λE, which in turn influence turbulent transport of heat and moisture in the ABL. This also changes cloud coverage, cloud microphysics, and the capacity to move air masses from the ABL into the free troposphere.^34^

Finally, BVOCs emitted by vegetation and other biogenic‐origin substances such as pollen also play a critical role in influencing radiation, temperature, and local precipitation patterns (Figure 1). BVOCs, in particular, contribute to the formation of aerosols and cloud condensation nuclei, affecting cloud properties, the partitioning of direct and diffuse incoming shortwave radiation (including PAR), and the Earth's atmosphere radiative balance.^35^ These compounds may lead to the cooling of the atmosphere by increasing cloud reflectivity, yet they may also cause warming, since they enhance the lifetime of CH_4_ and contribute to the formation of O_3_ and other greenhouse gasses in the presence of nitrogen oxides.^36^, ^37^ Their emission is highly species‐dependent, and it remains unclear the degree to which a high species diversity influences BVOC concentrations.^38^ Overall, the full causal chain from  ecosystem characteristics to the release and effects of BVOCs on cloud formation and temperature is highly complex. In fact, not only the total BVOC concentration but also the relative abundance of specific BVOC molecules and the background atmospheric chemistry are important.^37^ Field research has evidenced that in boreal forests under stress, increasing BVOCs promotes cloud formation,^39^ and that certain pollen types can have analogous influences on clouds.^40^

In summary, vegetation regulates the amount of moisture available for convective cloud formation through transpiration and interception loss. Cloud formation also depends on turbulence, which transports heat and moisture, promotes ABL growth, and is also directly controlled by the state of the ecosystem. Furthermore, vegetation emits BVOCs and pollen, enhancing the abundance of condensation nuclei that are key to forming clouds and rain droplets. Clouds and aerosols, in turn, regulate the amount of radiation and the fraction of diffuse PAR reaching the surface, further illustrating the complex feedbacks between vegetation, the ABL, and cloud formation. Predicting upcoming weather as well as future climate conditions requires an accurate understanding of this two‐directional vegetation–atmosphere coupling. In particular, it remains unclear how these bidirectional effects mutually interact during extreme conditions.^8^ Recent LES simulations have enabled the exploration of future scenarios of CO_2_ fertilization and warming—revealing changes in photosynthesis and ABL conditions—and have highlighted the need for integrated studies that consider soil, canopy, and atmospheric properties holistically.^34^ This highlights the importance of controlled experiments and comprehensive field campaigns to constrain and evaluate dedicated numerical simulations that explicitly resolve these two‐directional interactions to deepen our understanding of this complex, coupled system.^10^, ^41^

## INFLUENCE OF VEGETATION ON ATMOSPHERIC DYNAMICS

Vegetation exerts a substantial influence on atmospheric dynamics, impacting wind patterns at a wide range of spatial scales (Figure 1). In fact, vegetation has been proposed as a key factor in processes such as the slowdown of global near‐surface winds,^42^ moisture convergence over forests,^43^ and the expansion of the Hadley cells.^44^ Furthermore, it is known to impact mesoscale circulation by modifying thermal and moisture contrasts with surrounding regions (Figure 1). Vegetation may enhance or dampen sea breezes and valley–mountain flows,^45^ and even influence monsoon intensity.^46^ Moreover, since the distribution and characteristics of vegetation affect the large‐scale thermal properties over land, vegetation may influence synoptic‐scale atmospheric pressure patterns, and, therefore, the location and intensity of semi‐permanent highs and lows, which are critical features in our Earth's climate system (Figure 1). For example, large forested areas can increase near‐surface atmospheric temperature and humidity, potentially weakening (strengthening) high‐pressure (low‐pressure) systems.^47^ The influence of vegetation on atmospheric dynamics, from local to global scales, underscores the critical role of terrestrial ecosystems in climate regulation, helping explain the correlation between global ecosystem distribution and climate patterns.^3^ Nonetheless, some of the findings regarding the influence of vegetation on circulation patterns remain controversial, as discussed below.

The term “global stilling” refers to the observed reduction in terrestrial near‐surface wind speeds measured in recent decades over land.^48^ This phenomenon contrasts with the expected increase in wind activity in a warming world, and with the observed increasing trends in wind speed over the oceans.^49^ Nevertheless, increased near‐surface wind speeds at higher latitudes have also been reported in both hemispheres, pointing to important regional variations over land.^50^ The stilling trends have been linked to increases in surface roughness, primarily due to vegetation growth.^42^ Nonetheless, subsequent analysis based on near‐surface wind speed observations along with a conceptual boundary layer model attributed wind speed changes to changes in roughness, but the precise drivers, such as urbanization or forestation, were less clearly defined.^51^ In fact, later work using Earth System Models to isolate the response of near‐surface wind speed to increases in LAI, found that enhanced LAI was not a dominant driver of global stilling.^52^ Finally, it should be noted that the rate of stilling has seemingly weakened or even reversed in recent decades,^53^ and that land‐cover change is only one out of multiple factors that explain global stilling trends and their reversal.^54^ These insights highlight the complex and uncertain role of vegetation in shaping local and regional wind patterns, underlining the need for enhanced observational capabilities and modeling efforts to understand the drivers behind wind changes.

At the planetary scale, Hadley circulation transports heat and moisture from the equator toward the subtropics, shaping global weather patterns including storm tracks, subtropical high‐pressure systems, jet streams, and tropical monsoons. Multiple studies have reported a poleward expansion of the Hadley cells as our climate warms,^55^, ^56^ some of them relating this expansion to land–atmosphere feedbacks in drylands.^44^, ^57^ As drylands expand and vegetation diminishes, the resulting increase in surface albedo creates a feedback mechanism that may help expand the Hadley cells poleward. These vegetation‐driven changes in global circulation were first postulated by Charney,^58^ who hypothesized that a reduction of vegetation and consequent increase in albedo in the Sahel region would intensify the sinking of the Northern Hemisphere Hadley cell and perpetuate arid conditions. The expansion of the Hadley cells has important implications for regional water availability and has already been linked to drought intensification in parts of Australia.^59^ The influence of vegetation on these dynamics, particularly through changes in albedo, emphasizes again the complex interactions between terrestrial ecosystems and global atmospheric patterns. Understanding these vegetation‐driven changes in (sub)tropical circulation is crucial to accurately predicting and managing the role of drylands in their own expansion.^60^

Arguably, the most controversial thesis regarding the role of vegetation on global circulation is the biotic pump theory.^43^ This theory focuses on the importance of condensation‐induced atmospheric dynamics, positing that the large transpiration from forests, as well as the subsequent condensation over them, lowers the water vapor pressure in the lower atmosphere and leads to increased convergence of moisture from surrounding areas. Indeed, condensation affects atmospheric pressure through both latent heating and water vapor mass removal. While it is commonly accepted that the increased pressure due to latent heating dominates, Makarieva and Gorshkov^43^ emphasized the role of water vapor mass removal in atmospheric dynamics. As such, this theory implies that forests exert a profound influence on regional and global weather patterns by substantially enhancing moisture transport from oceans to land. This “moisture pull” of forests results in increased precipitation over terrestrial areas, and it also stabilizes and extends rainfall patterns. This theory has been heavily contested, yet seemingly without a definitive resolution.^61^, ^62^ Given the increased disturbance of forest ecosystems, understanding the mechanisms behind a potential biotic pump may be critical for predicting changes in global weather patterns and developing strategies to mitigate the adverse effects of deforestation on climate system dynamics.

In summary, the complex interplay between vegetation and atmospheric dynamics extends beyond local turbulence within the ABL, influencing major atmospheric weather processes and global circulation patterns. At the mesoscale, vegetation plays a potentially important role in regulating sea breezes and even monsoonal circulation. At a larger scale, vegetation may influence, for example, the subsidence associated with the Hadley cells and the location of semi‐permanent atmospheric pressure patterns. The global influence of vegetation is also seen in phenomena such as global stilling, with increased surface roughness due to vegetation growth potentially affecting near‐surface wind speeds. Moreover, the extensive transpiration of large forested areas can seemingly enhance moisture transport from oceans to land, stabilizing regional climates and modifying rainfall patterns. These dynamic interactions highlight the role of vegetation in atmospheric circulation. Advanced modeling and comprehensive observational strategies are essential to fully understand their importance and predict their implications in future climates.

## VEGETATION FEEDBACKS AND CLIMATE TRENDS

**FIGURE 2:**
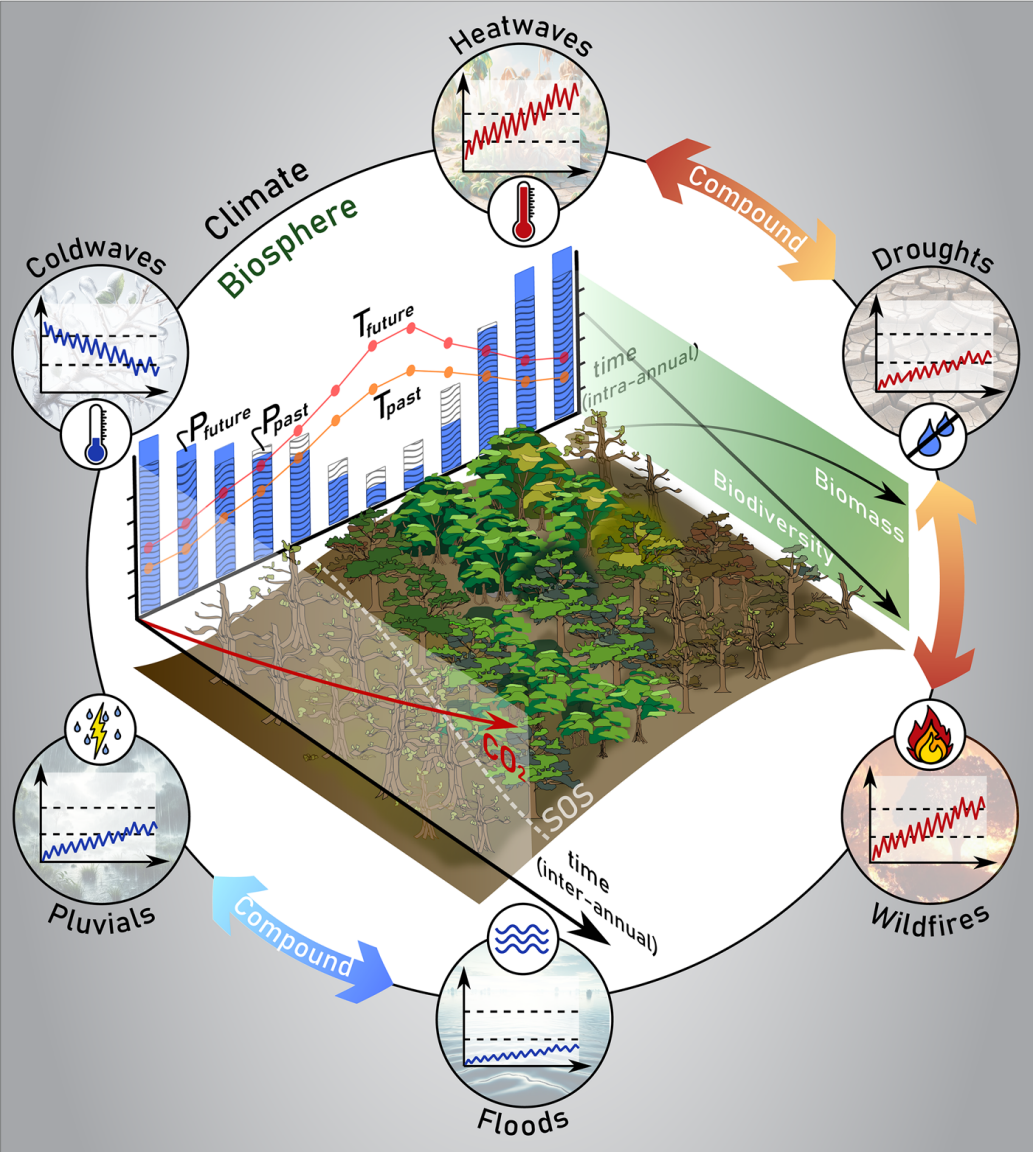
Interaction between vegetation and climate trends and extremes. Ongoing ecosystem trends, including a tendency toward larger biomass and lower biodiversity, regional succession and acclimation, or phenological trends—such as the earlier start of the season (SOS) and potential earlier senescence due to drought—are expected to influence temperature and precipitation trends and seasonality. Moreover, extreme climatic events not only impact ecosystems but may also be influenced by ecosystem dynamics and changes in vegetation structure and activity during these events. This is particularly the case of heatwave–drought–wildfire compound events, which are influenced by vegetation through multiple positive and negative feedbacks.

Understanding the processes by which vegetation influences the atmosphere across spatial scales is only a first, yet necessary, step in assessing how biophysical and biogeochemical feedbacks will shape temperature, precipitation, and other meteorological variables, as we move into the future. Climate perturbations associated with greenhouse gas (and aerosol) emissions and land use forcing have an influence on vegetation that spans from minutes to seasons and to millennia. This influence is, in turn, expected to either dampen (negative feedback) or amplify (positive feedback) the initial climate perturbations. Observational studies show that the recent tendency toward CO_2_‐richer and warmer atmospheres has already led to global greening in recent decades,^63^ largely due to extended growing seasons^64^ (Figure 2). This greening trend has contributed to an increase in global transpiration,^65^ which has partly been offset by water use efficiency increases due to reductions in stomatal conductance following CO_2_ fertilization.^66^ This reduced stomatal conductance has been proposed as a driver of precipitation changes in the tropics,^67^ global runoff increases,^68^ arctic warming,^69^ and the amplification of hot extremes.^70^ Likewise, the imprint of global greening on recent trends in temperature^71^ and precipitation^72^ has also been widely documented. While trends in water use efficiency remain highly uncertain,^66^ global greening trends have been showing signs of deceleration due to water limitation, among other factors,^73^, ^74^ and there is even a risk of trend reversal in future climates.^75^ In addition to water use efficiency and greening trends, the emission of CO_2_ also has repercussions on vegetation's phenology, influencing the senescence of leaves, altering surface albedo, the evaporation of water through transpiration and interception loss, the roughness of the ecosystem, and the entire carbon cycle.^64^ The impact of these phenological shifts on the climate system, particularly on precipitation and runoff patterns but also on temperature, remains an area of active research.^63^

In addition to global greening, water use efficiency, and phenological changes, climate change is also expected to affect ecological succession by changing ecological niches,^76^ in some cases favoring alien species that may transform autochthonous communities.^77^ Likewise, climate change can trigger large‐scale tree mortality due to a combination of plant heat and drought stress and, associated with this, more favorable conditions for fungi and/or beetle infestations.^78^ Large‐scale mortality events and changes in species composition due to adapted management and/or natural succession change the ecosystem's vertical structures and predominant plant functional traits (e.g., rooting depth, leaf mass, conduit density, and leaf nitrogen and phosphorus). Ecologists have argued that these expected changes in the diversity of plant functional traits (i.e., functional diversity) have an imprint on the functioning of ecosystems as a whole.^79^, ^80^ Studies have shown that prevalent plant functional traits and their diversity are closely linked to ecosystem‐scale functional properties, such as carbon uptake potential and water/light use efficiency.^81^ Changes in vegetation composition may occur progressively or abruptly as a tipping point,^6^ and the subsequent functional diversity may vary greatly. In any of these cases, multitemporal changes in terrestrial ecosystems are expected to feed back on regional, and most certainly global, long‐term climate trends.^82^

The recent IPCC AR6 report^15^ and CO_2_ assessments^16^ indicate that terrestrial ecosystems absorb approximately one‐third of anthropogenic CO_2_ emissions. Enhanced photosynthesis following CO_2_ fertilization, but also soil ecological processes,^83^ played an important role in increasing the terrestrial carbon sink in recent decades. The consequent dampening of the greenhouse effect implied a negative biogeochemical feedback of around –0.8 W m^−2^°C^−1^, yet with an uncertainty that is almost an order of magnitude higher over land than over the ocean.^84^ Should this be the magnitude of the feedback, it would equate to a buffering of global warming by around –0.5°C since pre‐industrial times; to give some perspective, that is, around half of the warming induced by the water vapor feedback, the strongest positive feedback in nature. However, around one‐third of the cooling associated with this CO_2_ biogeochemical feedback is thought to have been offset by the detrimental influence that the climate response to CO_2_ emissions had on photosynthesis.^15^ This mainly relates to CO_2_‐driven trends in soil moisture and temperature reducing gross primary production.^85^, ^86^ Finally, non‐CO_2_‐related biogeochemical feedbacks—such as those referring to the climate influence on BVOC emissions or land‐based release of CH_4_ and N_2_O—are reported by the IPCC AR6 as –0.16 W m^−2^°C^−1^, though with a high uncertainty.^15^ The influence of these biogeochemical responses on evaporation, precipitation, and runoff is even more uncertain and remains an outstanding research gap.^87^

Despite a historical focus on physical feedbacks—such as the snow albedo and atmospheric feedbacks (e.g., the cloud, lapse rate, and water vapor feedbacks)—the IPCC has traditionally concentrated preferentially on biogeochemical rather than biophysical feedbacks. Feedbacks associated with changes in leaf area, roughness, and/or soil moisture controls on transpiration have only been lightly touched upon by IPCC reports, likely due to the limited number of studies and large uncertainties. Therefore, the influence of these biophysical feedbacks on climate trends remains a crucial research gap.^9^, ^10^ Current estimates of the net biophysical feedback range from close to zero^82^ to +0.13 W m^−2^°C^−1^,^17^ while paleoclimatic approaches point to larger estimates around +0.3 W m^−2^°C^−1^.^11^ Given this limited evidence and high divergence among existing studies, the recent IPCC AR6 estimated the biophysical feedback as +0.15 ± 0.15 W m^−2^°C^−1^, assigning it a low confidence.^11^ Once again, the influence that changes in biophysical properties may have on the water cycle as we progress into the future remains even more uncertain.^87^ Finally, the influence of global greening on the surface albedo feedback—which is mostly dominated by snow and sea ice variability—is thought to be relatively limited,^11^ yet several studies have reported a warming associated with a shift from tundra to boreal forests in Northern Hemisphere high latitudes.^82^, ^88^

In addition to vegetation–climate feedbacks, the forcing associated with direct human perturbations—such as clearing land for agriculture, reforestation of abandoned farmland, and urbanization—has a direct and long‐lasting impact on terrestrial ecosystems and our climate system.^89^, ^90^, ^91^ The overall effects of anthropogenic land use and land cover changes may be comparable in magnitude to climate‐induced vegetation changes.^92^ Over decades to centuries, these land use changes drive successional shifts that alter community composition, ecosystem structure, surface energy fluxes, soil properties, carbon storage, and greenhouse gas emissions, thereby influencing trends in temperature^93^ and precipitation.^94^ Moreover, land cover changes have been highlighted as potential drivers of wind stilling over land,^42^ the expansion of the Hadley cells,^57^ and even of the slow‐down of the Atlantic Meridional Overturning Circulation.^88^ Nonetheless, the biophysical and biogeochemical processes associated with land use and land cover changes are crudely represented in global climate models, which may lead to inadequate projections of the influence of these changes on hydrology and climate trends.

The forcing associated with land cover changes and its influence on albedo has recently been estimated as –0.15 W m^−2^ since 1700 and –0.12 W m^−2^ since 1850, and likely resulted in a net global cooling of about 0.1°C since 1750.^95^ Moreover, the IPCC Special Report on Climate Change and Land concluded that there is robust evidence and high agreement that land cover and land use management exert an important influence on temperature, rainfall, and wind intensity at various spatial and temporal scales, through biophysical feedbacks.^96^ In light of this importance, intentional climate modification through land geoengineering strategies—such as reforestation or changes in land use—offers potential pathways for climate mitigation and adaptation.^97^ Such strategies should leverage vegetation's natural abilities to cool the local environment and/or enhance precipitation, thereby counteracting some of the adverse impacts of climate change.

## VEGETATION FEEDBACKS DURING HYDRO‐CLIMATIC EXTREMES

**FIGURE 3:**
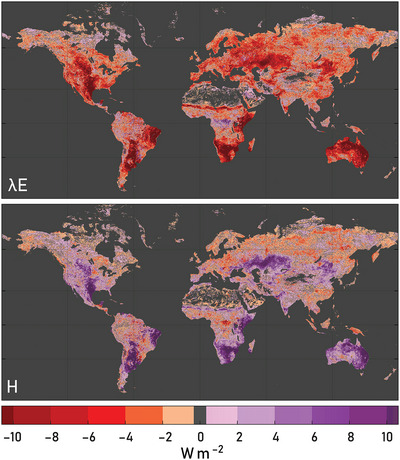
Impact of vegetation disturbances on the surface energy balance. Anomalies in latent (λE) and sensible (H) heat fluxes with respect to their local monthly climatology during times in which the leaf area index (LAI) anomalies drop below their 10th percentile (computed based on the pixel LAI climatology). The 1981–2023 period is considered. Data of λE and H come from GLEAM4 (https://zenodo.org/records/14056080), while LAI data come from GLOBMAP (https://zenodo.org/records/12698637).

As seen above, understanding the role of vegetation feedbacks in shaping long‐term climate trends over multiple spatial scales is crucial. Likewise, understanding the dynamic influence of vegetation feedbacks during extreme climatic events is critical for mitigating their societal and ecosystem impacts.^8^, ^98^, ^99^ Extreme events—such as droughts, heatwaves, coldwaves, wildfires, storms, and floods—directly affect water availability, agricultural productivity, ecosystem services, and human wellbeing.^100^, ^101^ Their regional exacerbation and more frequent concurrence as compound events are already felt around the world,^102^ highlighting the urgent need to understand their drivers for climate adaptation and resilience strategies.^103^ Since ecosystems are severely affected by climate events, and since consequent dynamic changes in vegetation state and activity will influence the surface energy balance, vegetation–climate feedbacks are expected to influence the evolution of these events.^101^ Figure 3 provides an overview of the impact of vegetation disturbances on the surface energy balance, by illustrating the anomalies in λE and H during times in which LAI anomalies drop below their 10th percentile (computed per pixel, considering the 1981–2023 period). Overall, lower‐than‐usual λE and higher‐than‐usual H are observed when vegetation is stressed, particularly in water‐limited regions with high hydro‐climatic variability. In high latitudes, low LAI events are often related to low radiation conditions, which lead to anomalously low values of both λE and H, while signals are more confounded in tropical forests where their variability is low and data tend to be more uncertain.

While the influence of vegetation on precipitation and moisture recycling has been studied for decades,^104^ its influence on the occurrence of pluvials (i.e., periods of excessive rainfall leading to abnormally wet conditions) has seldom been explored. Nonetheless, a recent study showed that more than half of the extreme rainfall during the 2021 European summer floods originated from plant transpiration and interception loss.^105^ In other words, while vegetation plays a crucial role in reducing overland flow and the risk of fluvial floods—by increasing the soil infiltration capacity, preventing erosion, and reducing sediment load in water bodies—it can also exacerbate storms due to enhanced moisture recycling, thereby increasing atmospheric moisture content, triggering convection, and inducing mesoscale circulation patterns (see previous sections). Nonetheless, a recent modeling experiment suggested that afforestation in Europe may decrease both the number and intensity of extratropical cyclones due to the increased surface roughness, although afforestation may enhance convective summer storms through increased transpiration.^106^ Overall, the influence of vegetation on flood occurrence due to its control of precipitation intensity needs to be considered in integrated assessments of land cover management aiming to reduce the risk of flood events, particularly in coastal regions where fluvial and pluvial floods are expected to be increasingly compounded with storm surges.^107^

Droughts and heatwaves are expected to aggravate and synchronize as we progress into the future (Figure 2).^100^ Current understanding suggests that similar, persistent large‐scale anticyclonic conditions (i.e., blocking highs) are behind the triggering of both events, while analogous land–atmosphere feedbacks—particularly through vegetation and soil moisture dynamics—play a crucial role in their intensification and propagation.^108^ Reduced evaporation from drying vegetation and soils increases H, potentially escalating heatwaves while also diminishing rainfall likelihood and further intensifying drought. In general, vegetation can modulate meteorological drought conditions through the moistening and warming of the atmosphere via transpiration, influencing local and regional humidity, convective stability, and precipitation. Healthy vegetation may mitigate meteorological drought by maintaining a certain level of moisture cycling within the ecosystem.^109^ Conversely, reduced vegetation activity as soils desiccate may lead to decreased transpiration, potentially exacerbating meteorological drought. In that sense, meteorological droughts can self‐intensify via land feedbacks.^110^ This is not only a local process, since the air advection from dry ecosystems reduces humidity and precipitation efficiency downwind, providing a mechanism for drought self‐propagation that can be dominant in semiarid regions.^110^

During heatwaves, vegetation cools the land surface by providing shade and consuming energy that would otherwise be available for sensible heating. As such, urban green spaces, including parks and green roofs, have been shown to reduce the urban heat island, making cities more resilient to heatwaves.^108^ Nonetheless, during the first phases of a heatwave, forested ecosystems can be substantially warmer than the surroundings due to their low albedo and more conservative use of water; then, as the event progresses, a sustained level of transpiration, enabled by this conservative use of water and their deeper roots, tends to lead to cooling compared to surrounding ecosystems.^111^ In other words, soil moisture–temperature feedbacks tend to be more positive during the onset, and less positive during the peak of heatwave events in forested areas. Moreover, these vegetation–climate feedbacks can lead again to teleconnected impacts, once downwind advection causes the spatial propagation of the event.^112^ Finally, the drying of vegetation combined with dry and hot atmospheric conditions has been shown to enhance the risk of wildfires during compound dry–hot events, a situation that is expected to aggravate in the future.^113^ Note that wildfire emissions then affect temperature, clouds, and rain, through the emission of greenhouse gasses and aerosols and changes in atmospheric stability.^114^

Changes in plant phenology also feed back on the occurrence of extreme climatic events. For instance, studies of European heatwaves show that delayed or weak growing season green‐up can amplify extreme heatwaves.^115^ In contrast, early and vigorous green‐up enhances transpiration and surface cooling, initially reducing the magnitude of warm temperature anomalies.^116^ However, at seasonal scales, some of these influences can be complex and seemingly counterintuitive. An earlier and more intense growing season, due to higher temperatures, can yield higher spring transpiration and lead to drier soils and vegetation in the summer, even without anomalies in precipitation.^117^ This has the potential to intensify summer heat and drought events and their consequences for ecosystems.^118^ Meanwhile, in addition to the importance of biodiversity for ecosystem resilience during climate extremes, its role in dampening the occurrence of certain extremes has also recently been highlighted.^8^, ^119^ Biodiversity—when understood as including genetic, functional, structural, and landscape diversity—can influence the capacity of ecosystems to buffer climate extremes, affecting processes like carbon sequestration, water retention, and overall ecosystem productivity. For instance, a higher functional diversity can enhance resilience to extreme conditions by providing a range of responses to stress and disturbance (“insurance hypothesis”).^120^ In the context of droughts and heatwaves, ecosystems with higher biodiversity may be better equipped to maintain function (e.g., transpiration and photosynthesis) due to the presence of species that can tolerate a range of conditions. This, in turn, can moderate local climate conditions and feed back into larger climate system dynamics.^8^ Here again, one should not overlook the influence of ecosystem functioning, including soil multifunctionality, as an actor in these feedback loops.^83^ The importance of biodiversity–climate and soil–climate feedbacks during extreme events emphasizes once more the critical role of vegetation–atmosphere interactions in the Earth's climate system, and the need to consider ecosystem dynamics in climate adaptation policies.^10^, ^91^

Despite substantial advances in our scientific understanding and capacity to observe and model vegetation properties and their changes over time, critical research challenges remain in disentangling vegetation's role during drought and heatwave events. Much of the current evidence still originates from experimental studies,^121^ and accurately representing ecosystem stress responses in terrestrial biosphere models remains challenging.^122^ Unsurprisingly, operational forecasts and climate models still struggle to capture the complexity of these vegetation–atmosphere interactions, particularly during extremes, leading to inaccuracies in early‐warning systems and climate extreme projections.^123^ The IPCC AR6 concludes—with medium confidence due to limited studies and evidence—that vegetation changes can amplify or dampen extreme events through changes in albedo and evaporation, influencing future trends in these events; it also concludes that urbanization increases the risks associated with extreme events by suppressing evaporative cooling and amplifying heatwave intensity.^124^ Moreover, the AR6 affirms that there is robust evidence that dry soil moisture anomalies favor summer heatwaves, and that part of the projected increase in heatwaves and droughts can be attributed to these feedbacks.^124^ The acknowledgment that vegetation feedbacks play a role in exacerbating or mitigating droughts and heatwaves paves the way for exploring climate engineering strategies aimed at modifying land surface conditions to attenuate these extremes. Measures such as altering crops albedo, modifying irrigation practices, implementing afforestation, and/or reforestation have been proposed.^125^, ^126^ However, the effectiveness of such strategies requires a comprehensive understanding of vegetation–climate interactions, highlighting the need for advanced models and observational tools to improve our predictive capabilities and develop sustainable mitigation approaches.

## CONCLUSION AND OUTLOOK

As climate change reshapes vegetation patterns, there is an urgent need to explore how these ecosystem changes will in turn affect climate, and ultimately impact biodiversity and ecosystem services. This review underscores the role of vegetation in regulating the atmosphere across all scales, from local effects in the ABL to impacts on global circulation patterns. The interactions discussed emphasize the importance of accurately representing biological processes and their coupling within climate models. Given the complexity of vegetation–climate feedbacks, an interdisciplinary approach is essential, integrating insights from biology, ecology, chemistry, hydrology, meteorology, and climatology. As we continue to gather experimental evidence from traditional manipulation experiments and long‐term monitoring efforts, refining our models and strategies through robust interdisciplinary research becomes increasingly important. Such refinement will enable better climate predictions, preparedness for future changes, and implementation of effective mitigation and adaptation strategies that leverage vegetation's natural regulatory capabilities. Several research gaps remain, including, but not limited to, (i) the role of vegetation in convective cloud formation and precipitation; (ii) the global magnitude of individual biophysical feedbacks; (iii) the effect of land cover changes on local and regional circulation; (iv) the biophysical and biochemical feedbacks associated with shifts in phenology; (v) the contrasting influence of CO_2_‐driven greening and water use efficiency increases on evaporation, precipitation, and runoff; and (vi) the interconnected impacts of biodiversity changes on climate trends and extremes.

Improving the understanding of these processes involves integrating satellite observations and ground‐based data. Refining process representation in fully coupled Earth system models is also essential, aiming not only for higher spatial and temporal resolution but especially for accurately capturing complex feedback mechanisms. Along those lines, the development of functional digital twins has become a central focus for most climate spheres. The goal is not only to create observation‐constrained models at unprecedented resolution but also to enable interactive simulations, allowing stakeholders to take anticipatory action.^127^ While digital twins for atmospheric and hydrological processes have achieved remarkable accuracy in representing cloud formation, evaporation, or river dynamics,^128^, ^129^ those specific to vegetation processes—especially biodiversity—remain at a conceptual stage.^130^ Consequently, the full integration of coupled vegetation–atmosphere processes into Earth system digital twins is not yet a priority. Finally, leveraging machine learning and hybrid modeling approaches, while respecting fundamental physical and biological principles, holds vast potential to foster our understanding of these complex interactions.^131^ Ultimately, understanding and predicting the feedback loops between vegetation diversity, ecosystem resilience, and climate stability is crucial for maintaining biodiversity and the health of our global ecosystems, and should remain a high research priority.

## AUTHOR CONTRIBUTIONS

D.G.M. led the analysis and writing of the first draft of the review. All authors contributed to the discussions and the writing and editing of the manuscript.

## COMPETING INTERESTS

The authors declare no competing interests.

### PEER REVIEW

The peer review history for this article is available at: https://publons.com/publon/10.1111/nyas.15286
